# Goiter and its associated factors among primary school children aged 6-12 years in Anchar district, Eastern Ethiopia

**DOI:** 10.1371/journal.pone.0214927

**Published:** 2019-04-04

**Authors:** Muzemil Muktar, Kedir Teji Roba, Bezatu Mengistie, Berhe Gebremichael, Adamu Belay Tessema, Meseret Woldeyohannes Kebede

**Affiliations:** 1 West Hararge Zone Health Office, Chiro, Ethiopia; 2 School of Nursing and Midwifery, Haramaya University, Harar, Ethiopia; 3 Department of Environmental Health Science, Haramaya University, Harar, Ethiopia; 4 School of Public Health, Haramaya University, Harar, Ethiopia; 5 Ethiopian Public Health Institute, Addis Ababa, Ethiopia; Boston University School of Medicine, UNITED STATES

## Abstract

**Background:**

Goiter is a major public health problem in Ethiopia. Even though there were studies done on goiter in Ethiopia, there was little evidence in the eastern part of the country. Therefore, the aim of this study was to assess the prevalence of goiter and its associated factors among school-age children in Anchar district of Eastern Ethiopia.

**Methods:**

A school based cross-sectional study was conducted from February 13 to 30, 2017. Multistage sampling method was used to select 418 children aged 6–12 years. Data were collected using a questionnaire. Children were examined for the presence or absence of goiter based on the criteria of the World Health Organization (WHO). Salt samples were tested using a rapid test kit. Data were entered to EpiData version 3.1 and exported to SPSS version 22.0 for analysis. Bivariate and multivariate logistic regression models were fitted; Crude Odds Ratio (COR) and Adjusted Odds Ratio (AOR) with 95% Confidence Interval (CI) were computed. Level of significance was determined at p-value less than 0.05.

**Results:**

The total goiter prevalence rate was 51.8% (CI: 46.9%, 56.8%). Father’s education (AOR = 1.87, CI: 1.06, 3.30), type of salt used (AOR = 2.09, CI: 1.13, 3.88), iodine level of salt (AOR = 2.77, CI: 1.11, 6.89), frequency of milk consumption (AOR = 3.65, CI: 1.63, 8.20), frequency of cabbage consumption (AOR = 7.74, CI: 4.48, 13.39), eating status of eggs (AOR = 3.16, CI: 1.54, 6.50), and eating status of dark green vegetables/fruits (AOR = 2.14, CI: 1.17, 3.93) were factors associated with goiter among school-age children.

**Conclusions:**

The total goiter prevalence rate was very high. Therefore, the health and education sectors of the study area should work hand in hand to improve the awareness of the community about goiter, iodized salt and iodine rich foods.

## Introduction

The hormones produced by the thyroid gland need iodine, as an essential component and these hormones are crucial for sustaining human life. Deficiency of iodine results in goiter which is the abnormal growth of thyroid gland, clinically detected by physical inspection and palpation [1, 2]. The prevalence of goiter increases with the severity of iodine deficiency and becomes endemic in populations where the intake of iodine is less than 10 microgram per day [3].

The involvement of both dietary and non-dietary factors besides iodine deficiency might interact in the genesis of thyroid enlargement [4]. Goiter is the most observable indication of iodine deficiency disorders, and it is a major public health problem for populations living in an iodine deficient environment [4–6].

Globally, the total goiter prevalence in the general population is estimated to be 15.8%, varying between 4.7% in America to 28.3% in Africa [7]. Moreover, nearly two billion people are at risk of iodine deficiency worldwide, while one-third lives in areas where natural sources of iodine are low [8, 9]. Regarding to the school children, the prevalence of goiter varies in different studies in the world, ranging 5.5% to 35.9% [10–19]. Furthermore, one-third of school children are estimated to have insufficient iodine intake where the highest magnitude is documented in Africa (39%) [20].

Ethiopia is among the iodine deficient countries in the world [21] where about 28 million people suffer from goiter and more than 35 million people are at risk of iodine deficiency [22]. A nationwide study conducted in the country revealed that the total goiter prevalence in school-age children was 39.9% [23]. Besides, other different studies showed that most of the districts in Ethiopia have high prevalence of goiter, varying 26.3% to 62.1% [24–31].

In addition to the depletion of iodine in the soil, the development of goiter in school-age children is significantly associated with age [24, 28, 29], sex [29, 30, 32], residence [32], maternal occupation [28], maternal education [27], source of drinking water [28, 32, 33], household wealth status [27, 28], consumption of goitrogenic foods [27, 32, 33] and iodine level of salt [27–30].

Ethiopia had endorsed the universal salt iodization program with the goal of reaching more than 90% coverage by 2015. Accordingly, two nationwide surveys conducted in 2015 and 2016 found that over 89% of the salt in the country contained iodine [34, 35]. However, only 26% of the household salts were adequately iodized (≥15 parts per million (ppm)) [35].

A goiter prevalence of 5% or more in school-age children is an indication of iodine deficiency in a population. As a result, the goiter rate in school-age children can be used to determine the severity of iodine deficiency in the population since they are easily susceptible to iodine deficiency, accessible as a study group and representative of society as a whole [2, 36].

Even though there were studies done on goiter in Ethiopia, its occurrence varies from area to area within the country. In addition, there was little evidence about goiter in the eastern part of Ethiopia. Therefore, this study was designed to assess the prevalence of goiter and its associated factors among primary school children aged 6–12 years in Anchar district of Eastern Ethiopia.

## Methods and materials

### Study setting and sampling

A school-based cross-sectional study was conducted among children 6–12 years of age from February 13-30/2017 in Anchar district, Eastern Ethiopia. The district is found at a distance of 390 kilometers to the east of Addis Ababa; capital city of Ethiopia. The district has 28 kebeles (*kebele is the smallest administrative unit in Ethiopia*) with a total population of 104,615 (53,354 females and 51,261 males) and total households of 21,795. The district has one preparatory school, three high schools and forty-four primary schools. The altitude of the district ranges between 1200 to 4500 meters above the sea level. The staple foods of the district are cereals and vegetables. The study populations were all school-age children (6–12 years) with their mothers/caregivers living in Anchar district. Children who were critically ill and living in the study area for less than six months were excluded from the study.

The sample size for the prevalence of goiter was determined using single population proportion formula considering the following assumptions: 95% confidence level, 5% margin of error and proportion of goiter among school-age children (50.6%) [24].

$$n=\frac{\left({Z_{\alpha /2})}^{2}p(1-p\right)}{d^{2}}=\frac{\left({1.96)}^{2}\times 0.51(0.49\right)}{({0.05)}^{2}}=384$$

The sample size for the associated factors was calculated in Open Epi online software assuming the following assumptions: 95% confidence level, 80% power, equal unexposed to exposed ratio (1:1), proportion of goiter among rural school-age children (58.6%) and urban school-age children (41.4%) [32]. This provided a sample size of 288.

Finally, the sample size that was calculated for the prevalence of goiter (384) was used in this study since it was greater than the sample size of the associated factors. A contingency of 5% was added for non-respondents, and resulted in a final sample size of 418.

**Fig 1:**
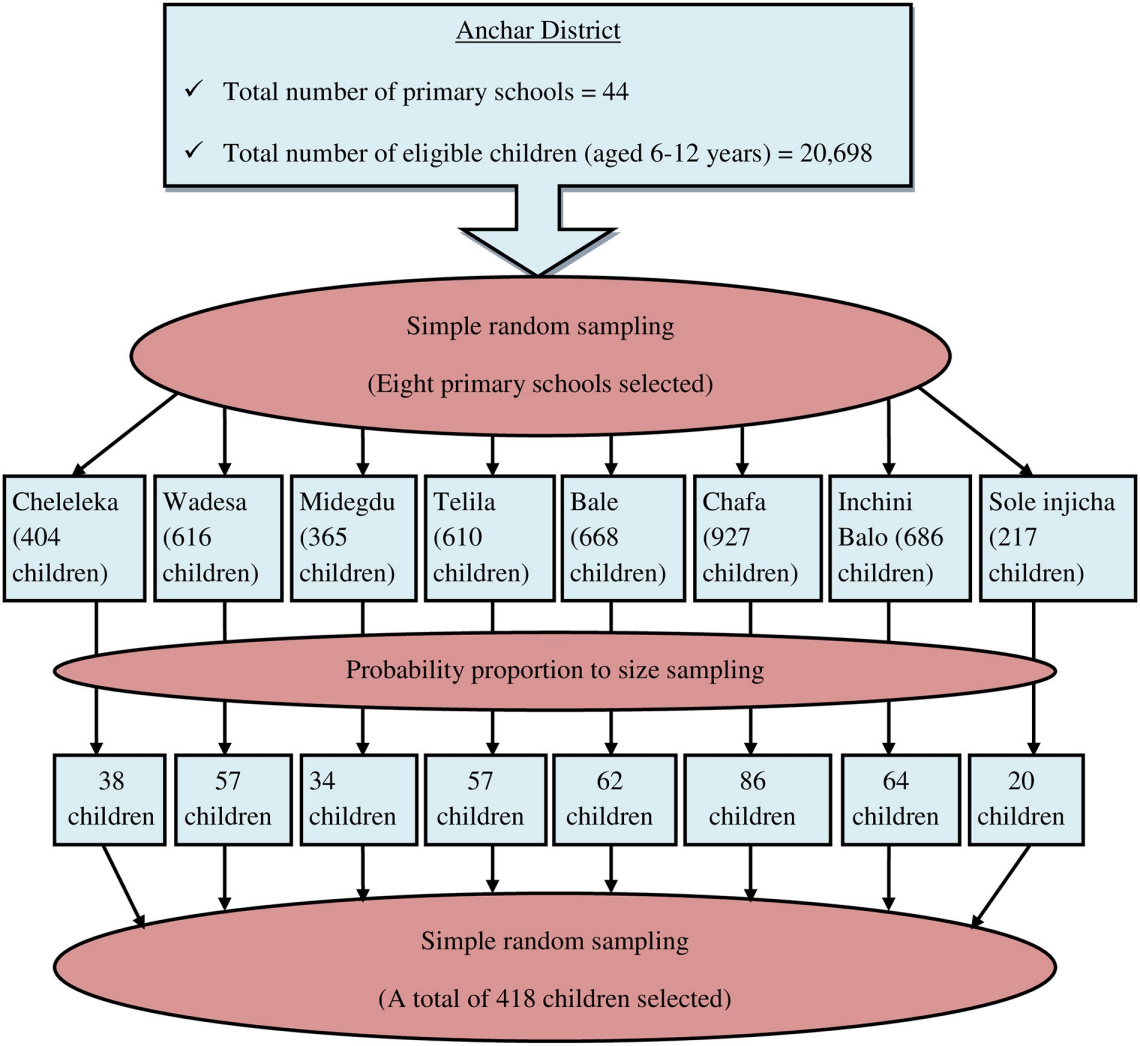
Sampling procedure among primary school children aged 6–12 years in Anchar district of Eastern Ethiopia, 2017.

Multistage sampling was used to select study participants. The first stage was school, whereas school-age child was the second stage. In the study area, 20,698 eligible children (6–12 years of age) were attending their education in forty-four primary schools in 2017. Eight of the primary schools, with 4493 eligible children, were selected by simple random sampling. The numbers and lists of eligible children were obtained from the directors’ offices of the selected schools, and sampling frames were created for each selected school. Then, the numbers of children to be included in this study were determined for each selected school by probability proportion to size sampling. Finally, the simple random sampling technique was employed to reach individual child (Fig 1).

### Data collection

Pre-tested and interviewer administered structured questionnaire was used to collect quantitative data. The questionnaire was prepared in English language and translated to Afan Oromo language, and back to English language. Ten experienced health officers and nurses, who had a BSc degree, undertook the interview after they had been trained for three days. Data were collected on socio-demographic characteristics, household assets and feeding habits of the children from themselves and their mothers/caregivers. Food frequency questionnaire and 24-hour recall methods were used to collect the data on the dietary habits of the children.

Checklists were used to collect data from anthropometric measurements, physical examination of the thyroid gland, and iodine measurements of household salt. The weight of the children was measured by an electronic digital balance. The balance had been readjusted to zero daily in the morning, and was checked whether it measures correctly by weighing an object of known weight. During weight measurement, heavy clothes, shoes, bags or any other material were avoided. The height of the children was measured by using portable anthropometer. The height measurement was done without shoes and heavy clothes. Regarding to the clinical assessment of goiter, physical examination was done on the thyroid gland by trained health officers as per the recommendation of WHO [2]. The results of the examination were reported as grade 0 for no palpable or visible goiter; grade 1 for palpable, but not visible goiter; and grade 2 for visible goiter.

All of the children were requested, one day before the interview, to bring salt and come with their mothers/caregivers. Then salt iodine content was measured using a rapid spot testing kit called MBIKITS INTERNATIONAL, which was made in India in June 2016. The test kit was produced with batch number of M 016 and was expired in November 2017. Finally, the iodine level of the salt was expressed in parts per million (sufficient ≥15ppm, medium <15ppm, and no iodine 0 ppm) [37].

### Data processing and analysis

After data collection, data were edited and cleaned; each questionnaire was checked for completeness and coded. Data were entered into computer using EpiData version 3.1 and the analysis was done using SPSS version 22.0. Categorical variables were described using frequency, percentage and tables. Continuous variables were assessed for normality and were described using summary measures. Anthropometric indices with z-score values were computed using WHO AnthroPlus software. Household wealth index was constructed by principal component analysis and the households were categorized into terciles; as poor, middle and rich.

Bivariable logistic regression analysis was used, and COR with 95% CI was computed to assess the association between each independent and the outcome variables. Then, variables with p-value < 0.25 were included to multivariable logistic regression analysis. The included independent variables were tested for multicollinearity using Variance Inflation Factor (VIF), and no significant (VIF > 10) collinearity was detected. Model goodness-of-fit was checked by Hosmer and Lemeshow test, and the final model was well fitted with the included variables (p-value = 0.22). Finally, the associated factors were identified by estimating AOR with 95% CI and statistical significance was declared at p-value < 0.05.

### Ethics approval and consent to participate

First, ethical clearance was secured from Institutional Health Research Ethics Review Committee (IHRERC) of the College of Health and Medical Sciences in Haramaya University. Then, official permissions were obtained from West Hararge zone and Anchar district health and education offices. Informed, voluntary, written and signed consents were obtained from each student’s mother/caregiver and school directors. In line with this, oral assent was obtained from the children themselves. The interviews and measurements were carried out privately in a separate room. Names and personal identifiers were not included in the questionnaire and checklist to ensure participants’ confidentiality.

## Results

### Socio-demographic and economic characteristics

Out of 418 sampled children, 407 children aged 6–12 years with their mothers/caregivers participated in the study (response rate = 97.4%). The reason for the non-response was that the children were unable to bring their mothers/caregivers for the consent and interview. Two hundred six (50.6%) of the children were males, with a mean age of 9.57 years (Standard Deviation (SD) = 1.38)). The majority (78.4%) of the children belong to Muslim families. Three hundred eleven (76.4%) of the children were living in rural areas and 201 (49.4%) were from the *Weinadega* ecological zone. Three hundred eleven (74%) of the children were from households with family size of five and above, and 139 (34.3%) were from poor households.

Regarding the socio-demographic characteristics of the mothers/caregivers, most of them were married (94.6%), with a median age of 34 years (inter-quartile range = 10). Three hundred forty seven (85.3%) of the mothers/caregivers were housewives by occupation and 297 (73%) were unable to read/write. In addition, 252 (61.9%) of the children’s fathers were unable to read/write **(**Table 1).

**Table 1:** Socio-demographic and economic characteristics of school-age children and their parents/caregivers in primary schools of Anchar district in eastern Ethiopia, February 2017 (n = 407).

Variables	Categories	Frequency	Percentage
Sex	Male	206	50.6
	Female	201	49.4
Child age (in years)	6–8	98	24.1
	9–12	309	75.9
Religion of the children	Muslim	319	78.4
	Orthodox	83	20.4
	Protestant	5	1.2
Ecological zone	*Kola*	158	38.8
	*Weinadega*	201	49.4
	*Dega*	48	11.8
Residence	Rural	96	23.6
	Urban	311	76.4
Marital status	Single	7	1.7
	Married	385	94.6
	Divorced	10	2.5
	Widowed	5	1.2
Maternal age (in years)	20–29	108	26.5
	30–39	210	51.6
	40–49	74	18.2
	50–59	10	2.5
	≥60	5	1.2
Maternal educational status	Unable to read and write	297	73.0
	Able to read and write	56	13.8
	Primary (grade 1–8)	35	8.6
	Secondary (grade 9–12)	6	1.4
	College and above	13	3.2
Mother’s occupational status	Housewife	347	85.3
	Government employee	17	4.2
	Private employee	22	5.4
	Day laborer	20	5.1
Educational status of father	Unable to read and write	252	61.9
	Able to read and write	64	15.7
	Primary (grade 1–8)	57	14.0
	Secondary (grade 9–12)	9	2.3
	College and above	25	6.1
Family size	Less than five	106	26.0
	Five and above	301	74.0
Household wealth index	Poor	139	34.3
	Middle	126	31.1
	Rich	140	34.6

### Mothers’/caregivers’ awareness of health and nutrition

Out of the 407 children’s mothers/caregivers included in this study, 248 (60.9%) of them had no information on health or nutrition. Two hundred sixty four (64.9%), 90 (22.1%), and 302 (74.2%) of the mothers/caregivers had no awareness of a balanced diet, iodine rich foods and iodized salt, respectively. Besides, 150 (36.9%) of the mothers/caregivers believe that every salt contains iodine, and 320 (78.6%) of them do not buy/use iodized salt for their families. Regarding the information on goiter, 323 (79.4%) of the mothers/caregivers had heard about goiter. However, 123 (30.2%) and 243 (59.7%) of them had no awareness of the major causes and prevention methods of goiter, respectively.

### Dietary habits of the children

The average meal frequency of the children was two times per day (SD = 0.63). Cabbage, legumes and oils/fats were reported to be the most frequently consumed food items in the study area. One-hundred four (25.6%) of the children consumed cabbage twice per week. In addition, 243 (59.7%) and 305 (74.9%) of them consumed legumes and oil/fats daily, respectively. However, peanuts, milk, egg and sugar/honey were consumed less frequently. Concerning to the dietary diversity, 93 (22.91%) and 49 (12.0%) of the children consumed dark green vegetables/fruits and other vegetables/fruits during 24-hours prior to the study, respectively. Only 24 (5.9%), 55 (13.5%) and 59 (14.5%) of them consumed cereals/tubers, pulses and eggs, respectively. Generally, the mean dietary diversity score was 0.76 food groups (SD = 1.13), and 399 (98%) of the children consumed less than four food groups during the day prior to the study.

#### Iodine level of salt and nutritional status of the children

The iodine rapid test result showed that 250 (61.4%) of the sampled household salts were non-iodized. One hundred twenty three (30.2%) of the salt samples had inadequate levels of iodine (1–15 ppm) and 34 (8.4%) were adequately iodized (≥15 ppm).

Concerning the nutritional status of the children, 35 (8.6%) and 110 (27%) of them were severely and moderately stunted, respectively. Besides, 13 (3.2%) and 49 (12%) of the children were severely and moderately underweight, respectively (Table 2).

**Table 2:** Nutritional status of primary school children aged 6–12 years and the iodine level of households’ salt in Anchor district of eastern Ethiopia, February 2017 (n = 407).

Variable	Categories	Frequency	Percentage
Iodine level of household salt (in ppm)	0 ppm	250	61.4
	1–15 ppm	123	30.2
	≥15 ppm	34	8.4
Height-for-age (z-score)	Severe stunting (<-3)	35	8.6
	Moderate (-3 to -2)	110	27.0
	Normal (>-2)	262	64.4
BMI-for-age (z-score)	Severe underweight (<-3)	13	3.2
	Moderate underweight (-3 to -2)	49	12.0
	Normal (-2 to 2)	345	84.8

### Prevalence of goiter and its associated factors

The overall prevalence of goiter among school age children was 211 (51.8%, CI: 46.9%, 56.8%). The magnitude of grade one goiter was 171 (42%, CI: 37.1%, 46.9%) while grade two was 40 (9.8%, CI: 7.4%, 12.8%).

Bivariate and multivariate analyses were done in the binary logistic regression to identify factors associated with goiter. Accordingly, the father’s level of education, buying/using iodized salt, frequency of milk and cabbage consumption, eating status of egg and dark green vegetables/fruits, and the iodine level of household salt were identified as associated factors of goiter among school-age children.

Children whose fathers did not attend formal education were 1.87 times more likely to have a goiter (AOR = 1.87, CI: 1.06, 3.30). The odds of goiter was 2.09 times higher among children whose families do not buy and/or use iodized salt (AOR = 2.09, CI: 1.13, 3.88). Likewise, children whose families salt with inadequate levels of iodine (<15 ppm) were 2.77 times more likely to have goiter (AOR = 2.77, CI: 1.11, 6.89).

The occurrence of goiter was 3.65 times higher among children who do not consume milk at all, compared to those who consume three times and above a week (AOR = 3.65, CI: 1.63, 8.20). Similarly, children who do not consume egg were 3.16 times more likely to develop goiter (AOR = 3.16, CI: 1.54, 6.50). On the other hand, the occurrence of goiter was 7.74 times higher among children who consume cabbage at least once a week (AOR = 7.74, CI: 4.48, 13.39). In line with this, children who consume dark green vegetables/fruits as a food were 2.14 times more likely to develop goiter (AOR = 2.14, CI: 1.17, 3.93) (Table 3).

**Table 3:** Factors associated with goiter among school children aged 6–12 years in Anchar district of Eastern Ethiopia, February 2017 (n = 407).

Independent Variables	Goiter Status of Children		COR (95% CI)	AOR (95% CI)
	Yes	No		
Child age in years				
6–8	41 (41.8%)	57 (58.2%)	1.00	1.00
9–12	170 (55.0%)	139 (45.0%)	1.70 (1.07, 2.69)*	1.59 (0.92, 2.75)
Educational status of fathers				
No formal education	175 (55.4%)	141 (44.6%)	1.90 (1.18, 3.05)*	1.87 (1.06, 3.30)*
Has formal education	36 (39.6%)	55 (60.4%)	1.00	1.00
Mothers/caregivers were aware of health/nutrition				
Yes	63 (39.6%)	96 (60.4%)	1.00	1.00
No	148 (59.7%)	100 (40.3%)	2.26 (1.50, 3.39)**	1.53 (0.91, 2.56)
Mothers/caregivers buy and use iodized salt				
Yes	28 (32.2%)	59 (67.8%)	1.00	1.00
No	183 (57.2%)	137 (42.8%)	2.81 (1.70, 4.65)**	2.09 (1.13, 3.88)*
Frequency of milk consumption				
3 times and above a week	13 (26.0%)	37 (74.0%)	1.00	1.00
2 times a week	22 (52.4%)	20 (47.6%)	3.13 (1.31, 7.51)**	2.87 (1.03, 8.02)*
Once a week	48 (55.8%)	38 (44.2%)	3.60 (1.68, 7.70)*	3.00 (1.23, 7.30)*
Not at all	128 (55.9%)	101 (44.1%)	3.61 (1.82, 7.15)**	3.65 (1.63, 8.20)*
Frequency of cabbage consumption				
At least once a week	183 (62.9%)	108 (37.1%)	5.33 (3.27, 8.67)**	7.74 (4.48, 13.39)**
Less than once a week	28 (24.1%)	88 (75.9%)	1.00	1.00
Eating status of dark green vegetables/fruits				
Yes	58 (62.4%)	35 (37.6%)	1.74 (1.09, 2.80)*	2.14 (1.17, 3.93)**
No	153 (48.7%)	161 (51.3%)	1.00	1.00
Eating status of eggs				
Yes	17 (28.8%)	42 (71.2%)	1.00	1.00
No	194 (55.7%)	154 (44.3%)	3.11 (1.70, 5.68)*	3.16 (1.54, 6.50)**
Iodine level of salt (in ppm)				
< 15	202 (54.2%)	171 (45.8%)	3.28 (1.49, 7.22)**	2.77 (1.11, 6.89)*
> = 15	9 (26.5%)	25 (73.5%)	1.00	1.00

* Statistically significant at p-value = 0.05–0.01,

** Statically significant at p-value < 0.01

## Discussion

The findings of this study assessed the prevalence and associated factors of goiter among school-age children in primary schools of Anchar district, Eastern Ethiopia. Accordingly, the prevalence of goiter in this study was 51.8% (CI: 46.9%, 56.8%). Father’s level of education, buying and/or using iodized salt, iodine level of household salt, frequency of milk and cabbage consumption, eating status of eggs and dark green vegetables/fruits were identified as independent predictors of goiter among school age children.

The present study showed that more than half of the children had goiter and according to WHO classification, the prevalence of goiter in the study area is very high [37]. This problem is happening in a country, where there is national mandatory universal iodization of salt and 89% of the household salts are iodized [34, 35]. This might be due to inadequate level of iodine in the salt.

The prevalence of goiter was higher in this study than other studies done in different parts of the world which ranges from 5.5% to 35.9% [10–19]. Similarly, the prevalence of goiter in this study was higher than different study reports in Ethiopia which showed a prevalence ranging from 26.3% to 62.1% [24–31]. However, the study reports from Shebe Senbo (59.1%) and Burie/Womberma (54%) districts revealed higher prevalence of goiter than this study [26, 38]. These all variations might be due to socio-demographic, socio-economic, cultural differences. In addition, the variations might also be due to differences in study scale, altitude and rainfall, feeding habits, access to iodized salt and iodine rich foods, and ways of cultivating the lands.

The study demonstrated that goiter was more likely to increase among school-age children whose fathers did not attend formal education. This finding was similar with the study done in Goba town, Southeast Ethiopia [24]. This could be explained as educated fathers are more likely to have more information, understand the education messages easily, more likely to be engaged in paid works and might receive lessons on child feeding and goiter at school.

The odds of goiter was higher among children whose families do not buy and use iodized salt. Likewise, children whose families salt with inadequate level of iodine were more likely to have goiter. This is supported by other different studies [27–30].

The occurrence of goiter in the current study was higher among children who do not consume milk at all. It was also observed that not consuming eggs increased the occurrence of goiter. This could be explained as eggs and dairy products are good sources of iodine. On the other hand, the occurrence of goiter was increased among children who consume cabbage at least once a week. In line with this, consumption of dark green vegetables/fruits was also significantly associated with the development of goiter among school age children. This was consistent with other studies [27, 32, 33]. These foods can decrease the iodine absorption and utilization in our body and in turn, increase the risk of iodine deficiency and goiter.

## Strength and limitations of the study

The strength of the study is that it was supplemented with salt iodine tests. However, since the study is cross-sectional, it might not show the temporal relation between the independent and dependent variables. There is a possibility that some of the responses might suffer from recall bias since the questions for the dietary habits were based on recall. Moreover, the result is not representative of the prevalence in the community, as it did not include children out of school.

## Conclusions

More than half of the school-age children included in this study had goiter. Moreover, more than half of the salt samples were non-iodized. Furthermore, the father’s level of education, buying and/or using iodized salt, iodine level of household salt, frequency of milk and cabbage consumption, eating status of egg and dark green vegetables/fruits were associated factors of goiter among school age children. Therefore, Anchar district health and education sectors should work together in disseminating messages to increase the awareness of the community on how to prevent goiter through the consumption of iodized salt, and iodine rich foods.

### Implications of the study

This study cannot be generalized to the national level and it may not influence the Ethiopian national food and nutrition policy. However, it can be used as an input, with other similar studies, in conducting systematic reviews and meta-analyses, thereby improving the national policy. In addition, it can be used as baseline information for further epidemiological and nutritional studies in similar settings. Moreover, the results of this study can help clinicians in decision making with regard to goiter diagnosis in the study area or similar settings.

## Supporting information

S1 Dataset The dataset from which the results of the study were produced. (SAV)

S1 Questionnaire The data collection tool (questionnaire) in English. (PDF)

S2 Questionnaire The data collection tool (questionnaire) in the original language; Afan Oromo. (PDF)

## Abbreviations

AOR: Adjusted Odds Ratio
CI: Confidence Interval
COR: Crude Odds Ratio
ppm: parts per million
SD: Standard Deviation
SPSS: Statistical Package for the Social Sciences
WHO: World Health Organization
