# Supplementary material for: Goiter and its associated factors among primary school children aged 6-12 years in Anchar district, Eastern Ethiopia
Source: PLoS One. 2019 Apr 4;14(4):e0214927. doi: 10.1371/journal.pone.0214927 (PMC6448855; doi:10.1371/journal.pone.0214927)
Supplement: S1 Questionnaire — (PDF) [file pone.0214927.s002.pdf]

English version of the questionnaire and checklist

Questionnaire identification number: __________________

Date of interview (DD/MM/YYYY): ______/____/______

Part-I: Socioeconomic and demographic characteristics of children and their parents/guardians

S.No	Question	Response
101	Child age (in completed years)	_____years
102	Child age (in completed months)	_____months
103	Child sex	1. Male<br>2. Female
104	Child religion	1. Muslim<br>2. Orthodox<br>3. Protestant<br>4. Other (specify)…
105	Ecological zone	1. Kolla<br>2. Woinadega<br>3. Dega
106	Residence	1. Urban<br>2. Rural
107	Total family/household size (in number)	____________
108	Marital Status of mother or caregiver	1. Single<br>2. Married<br>3. Separated<br>4. Divorced<br>5. Widowed
109	Age of mother	________years
110	Maternal educational status	1. Unable to read and write<br>2. Able to read and write<br>3. Primary (grade 1-8)<br>4. Secondary (grade 9-12)<br>5. College and above
111	Maternal occupation	1. Housewife<br>2. Government employee<br>3. Private employee/NGO<br>4. Day laborer<br>5. Unemployed<br>6. Other (specify) ______

112	Educational status of father	1. Unable to read and write<br>2. Able to read and write<br>3. Primary (grade 1-8)<br>4. Secondary (grade 9-12)<br>5. College and above
113	Do you have a radio?	1. Yes<br>2. No
114	Whose property is the house in which you live?	1. Our own<br>2. Rent<br>3. Free housing<br>4. Others (specify)
115	Does your household own any agricultural land?	1. Yes<br>2. No
116	Does your household own the following domestic animals? (More than one answer is possible)	1. Cows/bulls/oxen________<br>2. Horse/donkey/mules_____<br>3. Goats_____________<br>4. Sheep____________<br>5. Chickens_________<br>6. Beehives_________
117	Do you have a garden?	1. Yes<br>2. No
118	What is the main source of your drinking water?	1. Tap water in the compound<br>2. Bono<br>3. River/pond<br>4. Well/spring<br>5. Other (specify) ______
119	Does your household have latrine?	1. Yes<br>2. No
120	What is the main roof material of your house?	1. Rudimentary (Grass, Plastic sheeting)<br>2. Finished roof (corrugated iron)<br>3. No roof<br>4. Other (specify) _____

Part-II: Maternal /Caretaker's awareness or information on health and nutrition

S.No	Question	Response
201	Did you have information on health and nutrition?	1.Yes<br>2.No
202	Do you know what balanced/ diversified diet is?	1.Yes<br>2.No
203	Do you know iodine rich foods?	1.Yes

		2.No
204	Have you ever heard of iodized salt?	1.Yes<br>2.No
205	Do you think every salt contains iodine?	1. Yes<br>2. No
206	Do you buy and use iodized salt for your family?	1. Yes<br>2. No<br>3. Do not know
207	Have you heard of goiter?	1. Yes<br>2. No
208	What are the major causes of goiter?	1. Evil eye/evil spirit<br>2. Not eating adequate food<br>3. Drinking dirty water<br>4. Curse through/from family<br>5. I don't know
209	What are the food sources of iodine, if any?	1. Cereals<br>2. Fish/foods produced in sea area<br>3. Iodized salt<br>4. I don't know
210	How can goiter be prevented?<br>(More than one answer is possible)	1. Eating seafood<br>2. Eating iodized salt<br>3. Eating fish<br>4. Eating egg<br>5. Drinking holy water<br>6. Tattooing<br>7. Other (specify) _______________

Part-III: Feeding pattern/habit of children

S.No	Question	Response
301	How many meals does your child eat per day?	_________meals
302	Last week, how frequently did your child take?<br>(Put your answers in the provided space)	1. Milk ______________<br>2. Meat _____________<br>3. Egg ______________<br>4. Vegetables/fruits such as oranges after a meals ___________<br>5. Green leafy vegetables __________<br>6. Legumes based food ___________<br>7. Sweet potato ____________<br>8. Savoy cabbage (Kurunba) _________<br>9. Cabbage ("*gomman*") ________<br>10. Sugar/honey ___________<br>11. Oils/fats/butters ________
303	What is the cabbage eating frequency of your child?	1. Every day<br>2. 3x/week

		3. 2x/week<br>4. 1x/week<br>5. 1-2x/months<br>6. Never

Part-IV: Dietary diversity status of children

S.No	Question/characteristic	Response
401	Yesterday during the day and night, did your child eat any porridge or gruel made from grains and cereals?	1.Yes<br>2.No<br>3.I do not know
402	Yesterday during the day and night, did your child eat bread, pasta, rice, noodles, biscuits, cookies or any other food made from oats, maize, barley, wheat, sorghum, millet, or other grain?	1.Yes<br>2.No<br>3.I do not know
403	Yesterday during the day and night, did your child eat any food made from "*teff*", like injera, "*kita*", or porridge?	1.Yes<br>2.No<br>3.I do not know
404	Yesterday during the day and night, did your child eat any white potatoes, white yams, "*kocho*", cassava, or any other foods made from roots?	1.Yes<br>2.No<br>3.I do not know
405	Yesterday during the day and night, did your child eat any pumpkin, carrots, squash, or sweet potatoes that are yellow or orange inside?	1.Yes<br>2.No<br>3.I do not know
406	Yesterday during the day and night, did your child eat any dark green, leafy vegetables like kale, spinach or amaranth leaves?	1.Yes<br>2.No<br>3.I do not know
407	Yesterday during the day and night, did your child eat any ripe mangoes or papayas?	1.Yes<br>2.No<br>3.I do not know
408	Yesterday during the day and night, did your child eat any other fruits or vegetables?	1.Yes<br>2.No<br>3.I do not know
409	Yesterday during the day and night, did your child eat any liver, kidney, heart or other organ meat?	1.Yes<br>2.No<br>3.I do not know
410	Yesterday during the day and night, did your child eat any beef, fish, chicken or any other meat?	1.Yes<br>2.No<br>3.I do not know
411	Yesterday during the day and night, did your child eat any egg?	1.Yes<br>2.No<br>3.I do not know
412	Yesterday during the day and night, did your child eat any foods made from beans, peas, lentils or pulses?	1.Yes<br>2.No<br>3.I do not know
413	Yesterday during the day and night, did your child eat any nuts or seeds such as peanuts, sesame or sunflower seeds?	1.Yes<br>2.No

		3.I do not know
414	Yesterday during the day and night, did your child eat any cheese, yogurt, milk or other milk products?	1.Yes<br>2.No<br>3.I do not know
415	Yesterday during the day and night, did your child eat any foods made with oil, fat, or butter?	1.Yes<br>2.No<br>3.I do not know
416	Yesterday during the day and night, did your child eat any other solid or semi-solid food?	1.Yes<br>2.No<br>3.I do not know

Part-V: Physical and laboratory examination checklist for children

**S.No**	**Physical/laboratory Examination**	**Result**
601	Weight (in kg)	___________kg
602	Height (in cm)	___________cm
603	Goiter examination	1. Grade 0<br>2. Grade 1<br>3. Grade 2
604	Test the Salt with rapid test kit (in ppm)	1. 0 ppm<br>2. 0-15 ppm<br>3. ≥15 ppm<br>4. Not tested

Thank you!

Name of the interviewer ____________signature ______________date____________

Name of the supervisor ____________signature ______________date____________
