# Supplementary material for: Goiter and its associated factors among primary school children aged 6-12 years in Anchar district, Eastern Ethiopia
Source: PLoS One. 2019 Apr 4;14(4):e0214927. doi: 10.1371/journal.pone.0214927 (PMC6448855; doi:10.1371/journal.pone.0214927)
Supplement: S2 Questionnaire — (PDF) [file pone.0214927.s003.pdf]

Gaafannoo Afaan Oromoo

Ragaa tatamsa'ina quufa mormaa fi sababoota k'umsa isaa daaimman umrii barumsa mana barumsa sadarkaa 1ffaa aannaa Ancar, Godina Harargee Lixaa, oromiyaa, Etiyoophiyaa.

Mallattoo addaa gaafichaa ____________

Guyyaa gaafannoo ________/______/___________

Kutaa-I: Ragaa Waligalaa kan daa'imaa

Lakk	Gaaffii	Deebisaa
101	Umrii Daa'iimaa waggaadhaan	waggaa_________
102	Umrii ji'aan	Ji'aan ______________
103	Salaa Daa'imaa	1=Dhiira<br>2=Dhalaa
104	Amantaa daa'imaa	1. Muslima<br>2. Ortoodoksii<br>3. Proteestaantii<br>4. Kan biraa yoo jiraate ibsaa___________
105	Haala qilleensa naannoo jireenya daa'imichaa (maqa dhayi)	1. Baddaa<br>2. Badda daree<br>3. Gammoojjii
106	Iddoo jireenyaa	1. Mgaalaa<br>2. Baadiyyaa
107	Baayina warraa (lakofsaan)	________________
108	Haala fuudhaa/heeruma haadha yoking nama daa'imicha kunuunsuu?	1.Tan hin heerumin<br>2.Tan heerumte<br>3.Tan wajjiin hin jirre<br>4.Tan hiikatte/hiike<br>5.Tan du'aan adda baate/bahe
109	Umrii haadhaa yokiin nama daa'imicha guddisu?	Waggaa---------
110	Sadarkaa barumsa haadhaa ykn kunuunsituu	1.Barumsa ogummaa kan hin qabne<br>2.Barreessuu fi dubbisuu tan dandeessu

		3. sadarkaa tokkoffaa<br>4. sadarkaa 2ffaa<br>5.koolleejjii fi isaa ol
111	Hojii haadhaa	1. Haadha warraa<br>2. Hojjattuu mootummaa<br>3. Hojjattuu dhuunfaa<br>4. Hojjattuu guyyaa<br>5. Hojii dhabiyyee<br><br>Kan biro(ibsi__________
112	Sadarkaa barumsa haadhaa ykn kunuunsituu	1.Barumsa ogummaa kan hin qabne<br>2.Barreessuu fi dubbisuu tan dandeessu<br>3. sadarkaa tokkoffaa<br>4. sadarkaa 2ffaa<br>5.koolleejjii fi isaa ol
113	Raadiyoo niqabdanii?	1.Eeyyee<br>2. Lakki
114	Manni keessa galtan kun kan eenyuuti?	1. Keenya<br>2. Ni kireefanne<br>3. Kennaadha<br>4. Kan biro(ibsi___________)
115	Lafa qonnaa (dachii) niqabanii?	1. Eeyyee<br>2. Lakkii
116	Horii kana keessaa meeqa qaban?lakkofsaan	1. Loon/korma/qotiyyoo______<br>2. Farda/harree/gangee_______<br>3. Re'ee_________________<br>4. Hoolaa________________<br>5. Lukkuu_________________<br>Qafoo kannisaa___________
117	Qe'ee dubaa iddoo kuduraa fi muduraa itti qopheesitu ni qabdu?	1. Eeyyee<br>Lakkii
118	Bishaan dhugaatii eessaa argattan?	1. Bishaan bombaa mooraa keessaa<br>2. Bishaan bonbaa kan boonoo<br>3. Bishaan boollaa eegama/laga<br>4. Bishaan haroo/maddaa<br>Kan biro yoo jiraate(haa ibsamuu)_______

119	Mana fincaanii ni qabdanii?	1. Eeyye<br>2. lakki
120	Manni jireenyaa keessan maal irraa hojjatame	1. citaa<br>2. qorqoorroo<br>3. kan gubbaa hin qabne<br>4. kan biro ibsi___________

Kutaa-II: Haala waliigala odeefannoo Haadhaa yookin kunuunsituu fayyaa irratti

Lakk	Gaaffii	Deebii	
201	Haala sirna nyaataa fi fayyaa irratti hubannoo argattanii beektuu?	1.eyyee<br>2.lakkii	
202	Nyaata madaalamaa jechuu beektuu?	1.eyyee<br>2.lakkii	
203	Gosa nyaata Ayoodina gahaa qabu beektuu?	1.eyyee<br>2.lakkii	
204	Waa'ee Ashaboo Ayoodinii qabuu dhageessanii beektuu?	1.Eeyyeen<br>2.Lakki	Lakkii 306 tti darbi.
205	Ashaboon kamiyyuu ayoodina qaba jettanii yaadduu?	1. Eeyyeen<br>2. Lakki	
206	Ashaboo Ayoodina qabu bittanii bektuu / fayyadamuu?	3. Eeyyeen<br>4. Lakki<br>5. Qabaachuu fi dhabuu hinbeeku	eeyyee 309 tii dabra
207	Waa'ee dhibee morma quufaa dhageessee bekta?	1. Dhagaye<br>2. Hindhageenye	
208	Ka'umsi dhibee morma quufaa maali?	1. Budaa<br>2. Nyaata gahaa nyaachuu dhabuu<br>3. Bishaan qulqulluu hin tahin dhuguu<br>4. Sanyiidhaan	
209	Maddi iyoodinii maali?	1. Hin beeku<br>2. Qurxummii/ nyaata bishaan keessaa nyachuu<br>3. Ashaboo ayodinii qabu<br>4. Sanyii midhaanii	

210	Haala ittisa dhibee morma quufaa beektuu (deebii tokkoo ol qabaachuu danda’a)	1. Nyaata bishaan keessaa nyaachuun<br>2. Ashaboo iyodiinii nyaachuun<br>3. Qurxummii nyaachuu<br>4. Killee nyaachuun<br>5. Bishaan xabalaa dhuguu<br>6. Tumaa<br>7. Kan biraa (ibsi________)	

Kutaa-III: Gaaffii haalaa soorannaa daa’imaa ilaalchisee

Lakk	Gaaffii	Deebisaa	
301	Daa;ima kee guyyatti yeroo meeqa soorachiisaa jirta?	yeroo------------	
302	Torbaan darbe keessatti guyyaa meeqaaf nyaata kanneen daa’ima kee nyaachifte?fkn<br>Guyyarraa<br>Torbaanitti yeroo sadi<br>Torbaanitti yeroo lama<br>Torbaanitti yeroo takka<br>Gonkumaa	1.Huuloo(Goommanii)-----------<br>2.Hangudaaya--------------<br>3.Mixaaxis-------------<br>4.Dinnichaa-------------<br>5.Loozii---------------<br>6.Atara--------------	
303	Torbaan dabre daaimni kee goommana(raafuu) si’a meeqa nyaachifte?<br>1. Guyyarraa<br>2. Torbaanitti yeroo sadi<br>3. Torbaanitti yeroo lama<br>4. Torbaanitti yeroo takka<br>5. Ji’atti yeroo takkaa hanga lamaa<br>6. Gonkumaa	Eeyye=1, lakki=0 kan jedhu bakka duwwaatti guuta.<br>1._______________<br>2._______________<br>3._______________<br>4._______________<br>5._______________<br>6._______________	

Kutaa IV. Unkaa gaafannoo nyaata gosa adda addaa kan daa'imaa.

Guyyaa kaleessaatii fi halkan edaa nyaata armaan gadii kana daa'imni kee soorateeraa?		Yoo soorateera tahe sanduuqa armaan gadii keessaatti lakofsa tokko guuti yoo hin sooratin duwwaa guuti. 1= eeyye(soorateera),0= (Lakki(hin sooranne), 2 hin beeku
401	Nyaata kamuu waan akka shuroo/ marqaa, aximitii irraa hojjatamuu waan xaafiin allaa?<br><br>Nyaata kamuu waan akka serriifamii, fafa, milupa, bebilakii, mazar chozii ykn nyatta birroo kaa worshaan wolti makamee qopha'ee?	1= eeyye(soorateera),0= (Lakki(hin sooranne), 2 hin beeku
402	Daboo , pasta, ruzzaa, noodles, biskuttii , kuukisii ykn nyatota biro waan akkaa ajjaa, boqqoloo garbuu qammadii misingaa millet ykn waan akkaa midhaan biroo irra ka n hojjatame?	1= eeyye(soorateera),0= (Lakki(hin sooranne), 2 hin beeku
403	Nyaata kamuu xaffiirra hojjatame , kan akka budenaa,qixaa,ykn marqaa?	/-------/
404	Nyaata kamuu waan akka mixaaxish, bafuraa(bulla), qoccoo, cassava, ykn nyat kamiyyu kan hidhii nyaadhamuu?	1= eeyye(soorateera),0= (Lakki(hin sooranne), 2 hin beeku
405	Nyaata kamuu waan akka dubbaa, karroti, squash, ykn mixaxissaa waan akka booraa ykn birtukana fakatan?	1= eeyye(soorateera),0= (Lakki(hin sooranne), 2 hin beeku
406	Fudurawaan balii isaan magarisaa waan akka qosxaa, rafuu ?	1= eeyye(soorateera),0= (Lakki(hin sooranne), 2 hin beeku
407	Nyaata kamuu kan bilchattee waan akka mangoo,pappayaa?	1= eeyye(soorateera),0= (Lakki(hin sooranne), 2 hin beeku
408	Nyaata kamuu waan akka kudurafii fuduraa?	1= eeyye(soorateera),0= (Lakki(hin sooranne), 2 hin beeku
409	Nyaata kamuu waan akka tirruu, kallee onnee, foonii qamaa keessa ka akka garachaa fi marimaani?	1= eeyye(soorateera),0= (Lakki(hin sooranne), 2 hin beeku
410	Nyaata kamuu waan akka foon loonii,booyee, holla,re'ee, hilletii, [ykn foon bosoonuu ka akka borofaa/]?	1= eeyye(soorateera),0= (Lakki(hin sooranne), 2 hin beeku
411	Nyaata kamuu killee irra?	1= eeyye(soorateera),0= (Lakki(hin sooranne), 2 hin beeku
412	Nyaata kamuu kaa baqqeella ,atarii misiraa/lozzii ykn pulses irra hojatamee?	1= eeyye(soorateera),0= (Lakki(hin sooranne), 2 hin beeku
413	Nyaata kamuu kan akka ochollonii ykn seeds such as peanuts, sesame ykn(sufii)?	1= eeyye(soorateera),0= (Lakki(hin sooranne), 2 hin beeku
414	Nyaata kamuu kaa ananii ittitaa, ayibii ,ananii ykn sanyii ananii	1= eeyye(soorateera),0= (Lakki(hin sooranne), 2 hin beeku

415	Nyaata kamuu waan akka zayitaa, comaa ykn dhadha irra dalagamee?	1= eeyye(soorateera),0= (Lakki(hin sooranne),<br>2 hin beeku
517	Nyaata biro jabaataa(solid) ykn cinadhaan jabataa(semi solid) kan ta'e?	1= eeyye(soorateera),0= (Lakki(hin sooranne),<br>2 hin beeku

Kutaa V: Qorannoon quufa mormaa fi laboraatorii

Lakk.	Qorannoo	Bu'aa argame
501	Uffatina kiilograman(kg)	Kg_________
502	Dheerina daa'ima sentimitiraan (cm)	Cm_________
503	Qorannoo quufa mormaa gaggeessuu	1. Sadarkaa 0<br>2. Sadarkaa 1<br>3. Sadarkaa 2
504	Soogidda nyaataa qorachuu	1. 0ppm<br>2. <15 ppm<br>3.>15 ppm<br>4. hin qoratamne

Galatoomaa!

Maqaa Nama Uunkaa guutee---Mallattoo----------Guyyaa---------

Maqaa superviyzaraa ---Mallattoo----------Guyyaa---------------------
